# Resolving the Centipede’s Dilemma: external focus distance and expertise in applied, continuous skills

**DOI:** 10.1007/s00426-024-01951-y

**Published:** 2024-04-04

**Authors:** Stephen Banks, Peter Higgins, John Sproule, Ursula Pool

**Affiliations:** 1 University of Edinburgh, Edinburgh, UK; 2 University of Central Lancashire, Preston, UK

## Abstract

Research has reliably demonstrated that an external focus of attention during skill production enhances performance, retention, and transfer relative to an internal focus on movement mechanics. The optimisation of external focus points, across a range of contexts and performers, is important for effective skill production. Two studies were conducted evaluating the impact of external focus distance in an applied, continuous sports skill (kayak sprinting) with participants of two different expertise levels. In Study 1, using a within-participants design, recreational kayakers (*n* = 20) were timed sprinting 75 m in a surf ski under proximal external focus, distal external focus, and control conditions. The distal focus (on the finish) (29.75 s) was significantly faster than both other trials (*p*s < 0.001). The control condition (30.95 s) was significantly faster than the proximal focus (on the boat) (32.37 s) (*p* = 0.003). The effect size was large (*η*_p_^2^ = 0.55). In Study 2, specifically trained racers in sprint kayaks (K1s) (*n* = 16) were timed in a 100 m K1 sprint under the same three conditions as in Study 1. The control condition (28.96 s) was significantly faster than the proximal focus trial (29.83 s) (*p* = 0.02). The effect size was large (*η*_p_^2^ = 0.23). There was no significant difference between the distal focus trial (29.03 s) and the other conditions. These findings suggest that focus distance can play a pivotal role in continuous skills. Whilst recreational performers may benefit immediately from a distal focus, this might not be the case for specifically trained athletes. Further, a proximal focus on fitted, passive equipment may be detrimental to performance.

## Introduction

*A centipede was happy – quite!*

*Until a toad in fun*

*Said, "Pray, which leg moves after which?"*

*This raised her doubts to such a pitch,*

*She fell exhausted in the ditch*

*Not knowing how to run.*

Katherine Craster (1871)

Attentional focus effects during skilled performance have attracted interest historically. This is reflected in Craster’s (1871) poem imagining the detrimental impact on running if a centipede focuses attention on its (many) leg movements. Since the late 1990s, an increasing number of contemporary studies of attentional focus have repeatedly demonstrated a reliable link between an external focus and enhanced skill, relative to control conditions and focusing internally on the body and movement mechanics (e.g., Shea & Wulf, 1999; Wulf & Prinz, 2001; Wulf et al., 1998, 2001a). Considering the preponderance of skill learning and coaching which emphasises an internal focus (e.g., Durham et al., 2009; Porter et al., 2010; Van Vliet & Wulf, 2006; Yamada et al., 2022) these outcomes defied convention. However, work in this field has developed and, in line with questions posed historically, shows an external focus to consistently provide a robust learning and performance advantage across a range of movement skill situations.

In studies, an internal focus is created by asking participants to focus on part of their body involved in the required movement (e.g., the arms whilst swimming), whereas an external focus is placed on the desired movement effect (e.g., the water or the point being swum to) (e.g., Freudenheim et al., 2010; Stoate & Wulf, 2011). Using this approach, research has examined an array of skills and situations: An external (relative to an internal) focus results in superior movement efficiency, as may be measured via muscle electrical activity (e.g., Lohse et al., 2010), force generation (e.g., Marchant et al., 2009), muscular endurance (e.g., Marchant et al., 2011), respiratory efficiency (e.g., Schücker et al., 2016) and enhanced movement effectiveness such as improved success, reliability and precision (e.g., Abdollahipour et al., 2015; Mornell & Wulf, 2019). These benefits have not been bound by the activity, level of expertise, age, or infirmity (e.g., Abdollahipour et al., 2019; Chiviacowsky et al., 2010; Flores et al., 2015; Porter et al., 2015) and have been apparent in a broad range of physical skills from numerous sports as well as the playing of musical instruments and activities associated with physical therapy and rehabilitation. (see Marchant, 2011; Wulf, 2013 for reviews).

Focusing internally on movement mechanics demonstrably interferes with automaticity and the efficient activation of muscles, thus detrimentally affecting movement outcomes (e.g., Allingham & Wöllner, 2021; Lohse et al., 2011). The Constrained Action Hypothesis (McNevin et al., 2003; Wulf et al., 2001b) proposed that an internal focus on motor movements constrains, and conflicts with, subconscious control mechanisms thus undermining performance and learning. Further, McKay et al., (2015) highlight that an internal focus may increase awareness of the self, which in turn may lead to “micro choking episodes” due to the increase in conscious control over movement (see also Wulf & Lewthwaite, 2010). An external focus, conversely, has the apparent advantage of removing or reducing a damaging self-focus and internal focus. It also avoids distracting and competing attentional cues by maintaining a focus on the intended and required task outcome. Such benefits are manifest in faster, more accurate and reflexive movements (e.g., Kuhn et al., 2021); more coherent and flowing performance (e.g., Harris et al., 2019) and more effective outcomes in dual and multiple tasks (e.g., Sherwood et al., 2020). These effects are apparent using both actual external focus points and imagined points (e.g., Singh & Wulf, 2022; Yamada et al., 2020). This increase in automaticity with an external versus an internal focus, leads to movement and performance advantages which appear relevant to all physical tasks, and which may make the difference between success and failure.

The benefits of focusing externally are well demonstrated, though the question of optimisation across a range of contexts is still the subject of investigations. One such avenue of study concerns external focus distance: Studies have shown that an external focus placed at a greater distance from, and more discernible from, the body and body movements (a distal focus), results in greater automaticity and improved performance compared to the use of an internal (on the body) or proximal external focus. Bell and Hardy (2009) found that experienced golfers performed under pressure more effectively with a distal focus (flight) versus a proximal focus (club) and an internal focus (arms); Porter et al., (2012) reported improved long jump performance with a focus on a distal point; McKay and Wulf (2012) found darts players benefitted from a distal focus (target) versus a proximal focus (flight). Banks et al., (2020) described a significant benefit to wild water kayak 100 m sprint speed with a distal focus on the finish versus either a proximal focus on the paddle or a control condition.

Whilst there now exists a significant body of research into attentional focus effects, most studies have used activities closed and discrete in nature, that is, they tend to be one-off, single movements performed in a predictable environment. This includes the use of movements which would not naturally be discrete and isolated but would be part of a serial or continuous whole. This, in turn, may make it more difficult to relate and generalise such results to a standard, complex activity. Full form activities, by their very nature, are more challenging to study and control, though one way in which we can explore such physical tasks is by using repetitive, cyclical skills. These continuous skills (e.g., cross country skiing, running, cycling, swimming, walking, canoeing) tend to involve moving through the environment to a future point, often at some distance from the start. Some work has been conducted using these types of activities, and the performance benefits have mirrored other work, in that an external focus has produced superior outcomes to an internal focus and control conditions (e.g., Stoate & Wulf, 2011, swimming; Schücker et al., 2009, treadmill running; Schücker et al., 2016, cycling). A key difference in continuous skills which are repeated over a long duration, is that there is more opportunity for the performer to be distracted from the optimum focus, or for a sub-optimal focus to deleteriously affect the outcome. With this in mind, it is pertinent to consider whether a consistently maintained external focus is beneficial over an extended period in a continuous skill.

The question of focus maintenance was addressed by Banks et al. (2020) who investigated focus effects in wild water kayak sprinting. Wild water kayaking is an open,^1^ continuous skill in which participants race down a course on a river whilst adapting appropriately to the fast-paced and constantly varying environment. The activity permitted the assessment of performance under different attentional conditions over a longer period in a normal form sporting context. The participants reported using a switching focus during a control trial sprint, though this was significantly bettered by a fixed external focus on the finish. This highlights that continuous skills tend to have a range of possible focal points, with distal points being potentially far away. It also makes the use of such activities valuable in understanding how best to optimise focus and may increase the field validity of such work—particularly important when coaches and their charges may often focus internally on movement mechanics, despite the growing body of support for external focus advantages (see Porter et al., 2010).

A further question is whether an external, and specifically distal external, focus is equally advantageous for performers of differing skill levels. Many attentional focus investigations have used novice participants, though several studies have shown that a distal external focus is advantageous for people of different expertise levels (e.g., Wulf & Su, 2007, golf; Bell & Hardy, 2009, golf; Porter et al., 2013, jumping; Ille et al., 2013, track sprinting; Porter et al., 2015, sprinting). This finding though, is not universal: Singh and Wulf (2020) reported high-skilled volleyball players passing a ball more accurately to a target on the wall with a distal external focus, whereas low skilled participants were more accurate with a proximal external focus. These differences in outcomes, and the importance of accurately identifying the advantages of an appropriate distal (or proximal) focus, for individuals of differing skill levels, mean further study is important.

The purpose of the present work was to further investigate performance differences when using varied external focus points in an applied continuous skill with participants of differing levels of expertise. Kayak sprinting was selected as the activity for this study, which we used in its closed skill form on placid water (i.e., with insignificant competing variables in the performance environment which participants would need to attend or adapt to). This permitted appropriate comparisons between expertise groups. We were interested to examine whether a distal focus on the finish would lead to superior performance relative to a proximal focus on the boat. A control condition was also included to provide a comparison with the kayakers’ self-selected, and perhaps familiar, attentional focus. Maurer and Munzert (2013) questioned whether familiarity with foci might yield superior outcomes compared to using directed, unfamiliar foci, though the majority of studies have found an external focus leads to the best performance outcomes irrespective of familiarity (e.g., Ille et al., 2013). On this basis we hypothesised that a distal external focus would produce faster sprint performance, versus a proximal focus, irrespective of expertise.

Participants in both our expertise groups performed kayak sprints under three conditions: distal focus, proximal focus, and a control trial with no specified focus. Potential effects of practice, fatigue and condition sequence were controlled by counterbalancing the trial order. Manipulation checks were conducted immediately after each sprint to ensure instructions had been adhered to.

## Method

### Study 1

#### Participants

Twenty experienced and active kayakers took part in this experiment with an age range of 19–70 years; mean age 55.4 years, *SD* = 12.8. Ten female and ten male participants were recruited from the Southern California paddling community; they had no knowledge of the study purpose. The participants were drawn from a range of paddle-sport backgrounds, though all had experience of multiple craft and environments, and all were competent to sprint in a surf ski on placid water (as assessed verbally in advance, as well as practically during the pre-trial familiarisation period). Seven participants had prior experience of paddling surf skis. Informed written consent was obtained from the participants prior to data collection. The study was approved by The University of Edinburgh’s research ethics committee and was conducted in accordance with the ethical standards prescribed in the 1964 Declaration of Helsinki.

#### Apparatus and task

**Fig. 1:**
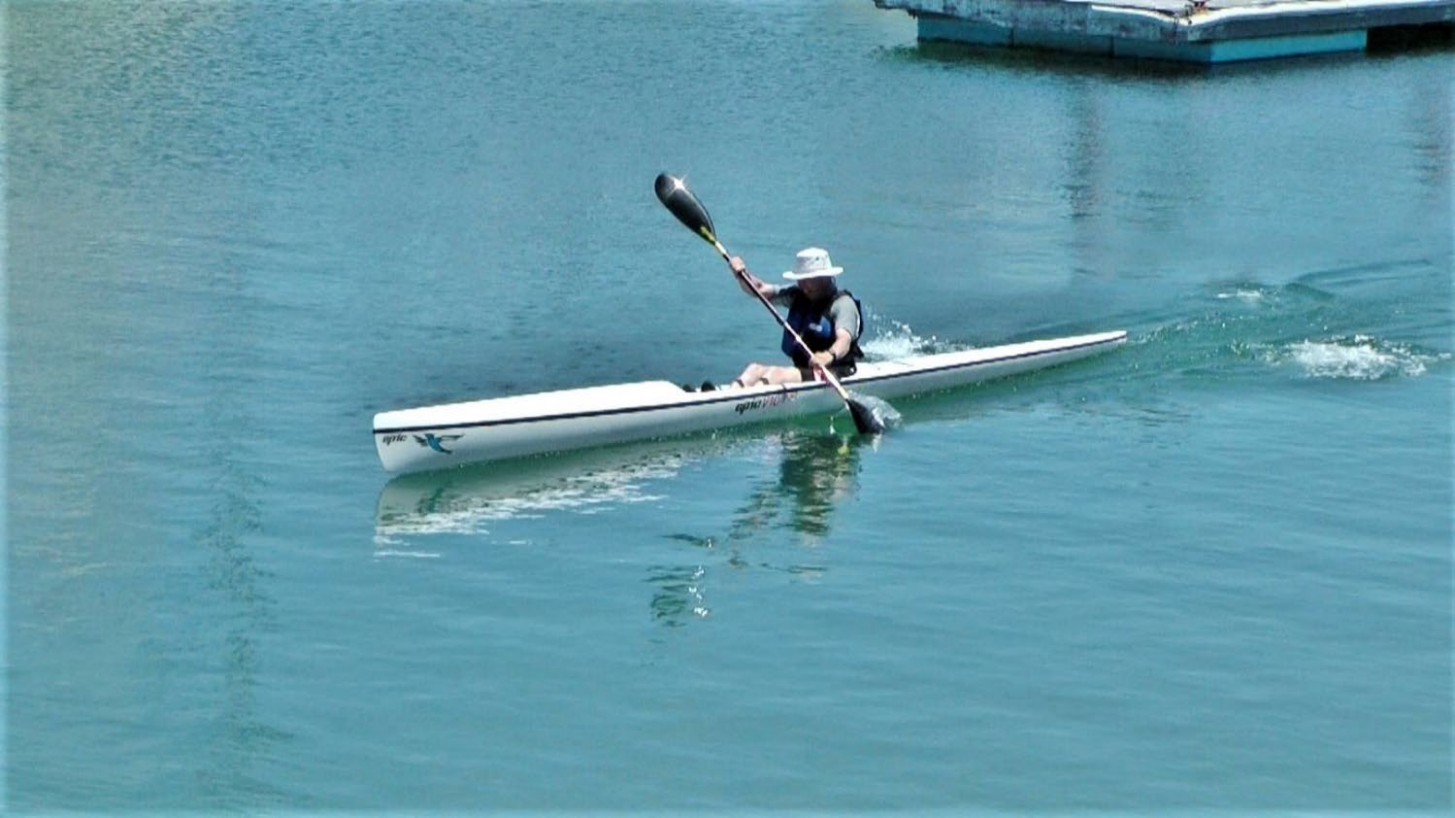
Sprinting towards the finish in the surf ski

The task comprised sprinting 75 m on calm water in a surf ski (Epic V10, adjustable for fit) (see Fig. 1). This distance was chosen, following pre-study trials with comparable volunteers, so that the study participants could maintain a full speed sprint for the longest time without being affected by fatigue. Surf Skis are long, narrow, sit-on-top craft which are capable of the highest speeds of any paddle-sport boat with the exception of sprint kayaks (K1s). The V10 used is 5.45 m in length with a beam of 0.45 m; steering is by means of a pedal operated rudder. The experiment was conducted at Quevira Basin Marina, San Diego, California, in an area free from disturbance that provided consistent placid water and climatic conditions. A video camera (Panasonic HDC TM 900) with a frame rate set to 25 Hz (0.04 s per frame) was used for accurate timing. This was aligned with a transit to form a timing point which the participants sprinted past. All participants were required to use the same paddle: an Epic, Greg Barton signature series carbon wing, adjustable for their size and handedness. All those taking part were asked to wear non-restrictive clothing suitable for a boat-based sprinting task and were provided with a buoyancy aid (personal flotation device) to wear.

#### Procedure

Participants attended and were tested individually. Each person was permitted sufficient time (approximately 20 min) to warm up and acquaint themselves with the boat and venue. One familiarisation trial was performed mirroring the subsequent trial conditions: participants were asked to sprint the length of the identified course as fast as possible. No further instructions or information were provided.

Using a within-participants design, each paddler then completed three consecutive 75 m sprints, each under different conditions: proximal external focus, distal external focus, control. The condition order was counterbalanced to control for fatigue, practice etc. (i.e., proximal–distal-control, distal-control-proximal, control-proximal–distal). Visual focus was controlled in all trials by requiring the participants to look at the same fixed point: an obvious post on the end of a pier beyond the finish and directly in their sight line as they sprinted down the course. In all trials the participants were asked to sprint to a defined point (‘the finish’) beyond the transit to ensure they were at full speed as they passed this timing point. In the control condition no additional instructions were provided (‘Sprint down the course as fast as you can’). In the proximal focus condition, participants were required to focus on the boat (‘Sprint down the course as fast as you can. Think only about the boat for the whole sprint’). In the distal focus condition, the participants were asked to focus on the finish (‘Sprint down the course as fast as you can. Think only about the finish for the whole sprint’). A check was conducted to ensure the instructions were clear and the necessity of keeping to them was understood.

At the start, the surf ski was lightly held until the participant was ready to go. The timing was calculated by subsequent frame-by-frame analysis of the video, from the point at which the paddle blade touched the water on the first stroke, until the point at which the bow of the boat reached the transit. After each sprint, the paddlers were helped from the boat and seated on the bank. Manipulation checks were conducted to establish what percentage of time they had looked at the visual point (post) in each trial. Following the control trial, the participants were asked what they had focused their attention on. After the proximal and distal focus condition trials they were asked what percentage of the sprint they had concentrated on the boat and finish respectively. There were approximately 10 min between sprints to gather information and permit recovery.

#### Data analysis

A one-way repeated measures ANOVA was employed to analyse the sprint times. All post hoc tests were treated with a Bonferroni adjustment. Mauchly’s test indicated that the assumption of sphericity had been violated, χ^2^(2) = 7.98, *p* < 0.05, therefore degrees of freedom were corrected using Greenhouse–Geisser estimates of sphericity (ε = 0.74). The attentional focus reports from the participants after the control trial were classified as distal external focus (e.g., on the post), proximal external focus (e.g., on steering; on the paddle stroke), internal focus (e.g., on arm action; on foot pressure) or an unspecified focus.

### Results

#### Sprint times

**Fig. 2:**
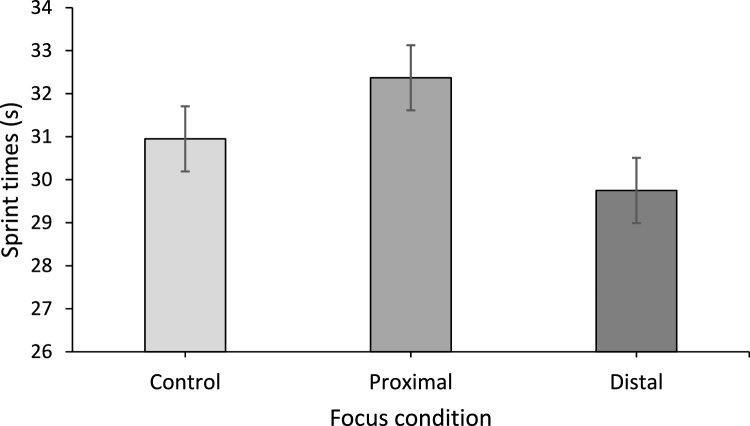
Mean sprint times for each experimental condition. Error bars represent standard errors

The mean sprint times for each condition are shown in Fig. 2. The distal external focus condition (29.75 s) was significantly faster than both the control (30.95 s) and the proximal external focus trial (32.37 s) (*p* < 0.001 in both cases). The control condition was significantly faster than the proximal external focus (*p* = 0.003). There was a significant main effect of attentional focus on performance speed: *F*(1.47, 28.0) = 23.30, *p* < 0.001, *η*_p_^2^ = 0.55. In the distal focus condition, participants completed the 75 m sprint, on average, 2.9 m ahead of the control trial and 5.9 m ahead of the proximal focus trial. The distal trial was therefore 3.7% and 7.9% faster respectively. The control trial was 3.2 m ahead of the proximal focus trial on average (4.4% faster).

### Manipulation checks

#### Visual focus

Participants kept their visual focus on the post as requested for all or almost all the duration of each trial: in the control condition they reported looking at the point 99.4% of the time, and in the proximal and distal focus conditions 100% of the time.

#### Attentional focus

In the distal and proximal focus trials, participants reported adhering to the prescribed focus all or most of the time. Specifically, in the distal trial 95.5% and in the proximal trial 99% of the time. The participants reported a range of focus points used in the control trial: five participants were unable to specify their predominant focus, seven reported a proximal focus, four reported an internal focus, and four reported a distal focus. The participants also reported frequently switching their focus during the control trial, between points which they deemed to be important.

### Study 2

#### Participants

Sixteen junior and youth K1 sprint kayakers from the San Diego Canoe & Kayak Team (SDCKT), took part in the study (seven females; nine males). The age range was 13–19 years, with a mean age of 14.8 years (*SD* = 1.9). SDCKT is an expertly staffed sprint kayak and sprint canoe racing team employing kayak coaches (the head coach was a former Olympic sprint kayaker and was senior coach for the USA junior national team), strength and conditioning coaches and a sports psychologist. All the participants followed detailed training programmes, regularly practised, and were specifically coached to paddle and race in K1 sprint boats. Written informed consent was obtained from all participants, their parents/guardians (for those under 18) and from SDCKT. The study was approved by The University of Edinburgh’s research ethics committee and was conducted in accordance with the ethical standards prescribed in the 1964 Declaration of Helsinki.

#### Apparatus and task

**Fig. 3:**
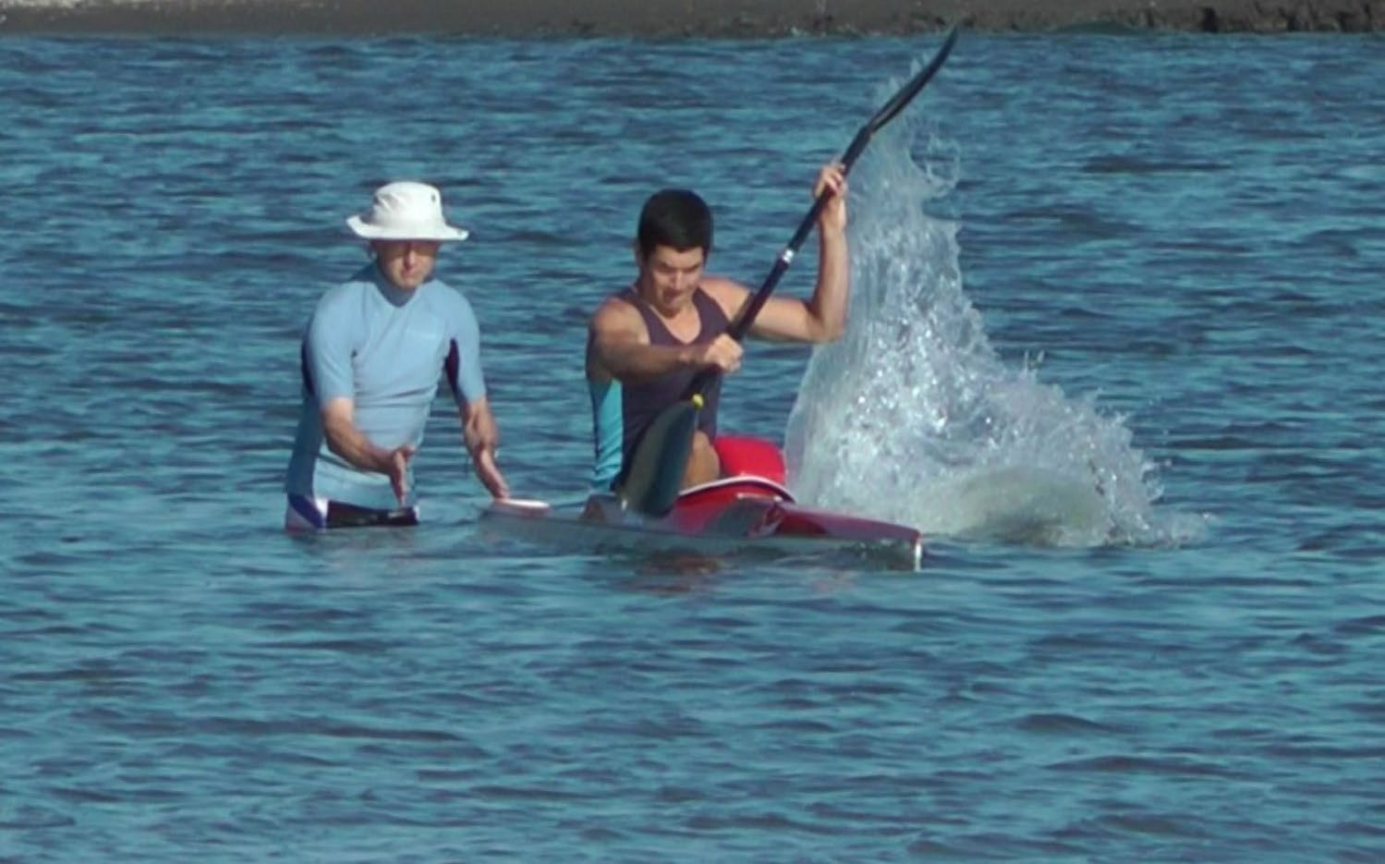
Participant at start of K1 sprint

The task entailed a 100 m K1 sprint on placid water at the SDCKT base in Enchanted Cove, Fiesta Island, San Diego, California (see Fig. 3). The distance was selected to provide a comparable length of sprint time to the participants in Study 1. K1 sprint racing kayaks are long and narrow with a rounded hull profile. Under International Canoe Federation (ICF) rules they must not exceed 5.2 m, though the width is not restricted—usually between 0.38 and 0.42 m. The boats are well-suited to travelling very quickly in a straight line and are the fastest class of kayak. The participants used their own, correctly fitted boats and paddles for the trials. A fixed buoy was used to mark the start; a transit aligned with a video camera (Panasonic HDC TM 900) was used as a timing point. A video frame rate of 25 Hz (0.04 s per frame) was used to ensure maximum accuracy.

#### Procedure

The participants were tested individually. They were initially invited to follow their normal warm-up routine (approximately 15 min) so as to be prepared for a series of three maximum effort sprints. Each paddler was then asked to sprint down the 100 m course as fast as possible to an identified point under three different experimental conditions (i.e., proximal external focus, distal external focus, control). The defined finish area was beyond the transit to ensure they passed this timing point at full speed. The trial order was counterbalanced across participants (i.e., proximal–distal-control, distal-control-proximal, control-proximal–distal). Additionally, participants were required to look at the same fixed visual point in all conditions (a pole on the jetty beyond the finish in their natural line of sight). In the control condition, no further instructions were provided. In the proximal focus condition, they were asked to focus on the boat. In the distal focus condition, the participants were required to focus on the finish. A check was made to ensure the instructions were clear and the necessity of complying with them fully understood.

At the start point, the rear of the kayak was held lightly (see Fig. 3) until the participant was ready to commence. Following each trial, participants paddled gently around the cove back to the start area in a wide arc to recover from the exertion. At this point manipulation checks were conducted: after the control trial, the participants were asked what they had focused their attention on during the sprint; following the proximal and distal trials they were asked what percentage of time they had concentrated on the boat and finish respectively. Additionally, after every sprint, the participants were asked what percentage of time they had looked at the visual point (pole). There were approximately 10 min between sprints.

#### Data analysis

Sprint times were analysed using a one-way repeated measures ANOVA. Bonferroni adjustments were made to all post hoc tests. Mauchly’s test indicated that the assumption of sphericity had been met, χ^2^(2) = 5.16, *p* > 0.05. Participants’ reports of attentional focus in the control condition were classified as distal external focus (e.g., on the water ahead), proximal external focus (e.g., on the paddle; on the boat), internal focus (e.g., on balance; on the feet, on their arm action), or other focus/comment (e.g., “felt solid”, “focused on fast”, “interfered with stroke rate”).

### Results

#### Sprint times

**Fig. 4:**
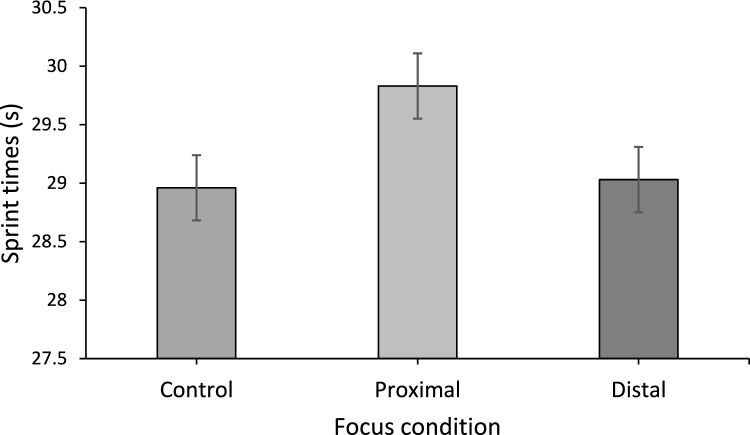
Mean sprint times for each experimental condition. Error bars represent standard errors

Mean sprint times for each condition can be seen in Fig. 4. The control condition (28.96 s) resulted in the fastest times. The distal condition yielded a similar time (29.03 s) to the control, whilst the proximal trial was the slowest (29.83 s). The attentional focus condition had a significant effect on the speed of K1 sprinting performance, *F*(2.0, 30.0) = 4.49, *p* = 0.02, *η*_p_^2^ = 0.23. Tests conducted post hoc indicated control trial times were significantly faster than those in the proximal focus trial. Relative to the control trial, the proximal condition sprint was 2.98 m behind at the finish, this equates to a 3% difference in speed. There was no significant difference between the control and distal trials or between the distal and proximal trials.

### Manipulation checks

#### Visual focus

Participants kept their visual focus on the pole as requested for all or almost all the duration of each trial: in the control condition they reported looking at the point (pole) 100% of the time; in the proximal and distal focus conditions 99.7% of the time.

#### Attentional focus

In the distal and proximal focus trials, participants reported adhering to the prescribed focus all or most of the time. Specifically, in the distal trial 97.8% and in the proximal trial 99.4% of the time. The participants reported a range of focus points used in the control trial: five participants were unable to specify their predominant focus, two reported a proximal focus (e.g., on the paddle, pedals or boat), four reported an internal focus (e.g., on their balance; arm cadence), and three reported a distal focus (e.g., on the finish area).

## Discussion

The present study aimed to extend understanding of attentional focus effects in continuous motor skills by manipulating the distance of external focus for both recreational and race-trained paddlers in a kayak sprinting task. Vision was directed at the same environmental feature in all conditions, ensuring that any differences in performance were attributable to attentional focus. Based on existing research findings, we hypothesized that a distal external focus would be more beneficial than a proximal external focus for both recreational and race-trained kayakers. The results reported above show that recreational kayakers completed a 75 m sprint on flat water faster when they were instructed to maintain a distal external focus of attention, compared with both a proximal external focus and no directed focus (control condition). This was consistent with our hypothesis. However, for K1 racers, instructions to maintain a distal external focus of attention during a 100 m sprint on flat water did not result in faster times than the proximal or control conditions.

The finding that a distal external focus benefitted performance for recreational kayakers is in line with previous findings in which a distal focus of attention was beneficial in motor skill performance (e.g., Banks et al., 2020; McKay & Wulf, 2012). This is consistent with explanations that suggest a benefit from shifting focus away from bodily movements (e.g., McNevin et al., 2003) and that focusing on the goal of the task (in this case the finish) may facilitate the whole-body movement necessary for accomplishing the outcome (Singh et al., 2022).

The instruction to adopt a distal focus did not result in a comparable increase in speed for race-trained kayakers. This was not in line with our hypothesis. As in previous studies with skilled performers, it may have been the case that the participants’ self-selected focus was simply too effective to be surpassed in a one-off directed trial. For example, Bull et al. (2022), found no benefit using an external focus of attention, compared with no directed focus, for skilled cricket players in a batting task. In an earlier study, Wulf (2008), found that world-class Cirque du Soleil acrobats performed a balance task more effectively in a control condition in which they self-selected their focus, compared with a directed external focus.

The control condition in the present study allowed us to compare performance under the prescribed focus conditions with the self-selected focus used by the participants. For both the recreational kayakers and the KI racers, instructions to maintain a proximal external focus resulted in significantly slower sprint times than the control condition. These findings are interesting, firstly because no difference was observed according to level of expertise: instructions to maintain a proximal focus disrupted performance for both groups of participants relative to the control condition. Also, in light of previous research (e.g., Freudenheim et al., 2010; Lawrence et al., 2019; Wulf et al., 1998) we anticipated that the instructions to maintain a proximal external focus might have improved performance compared to the control condition with no directed focus.

One potential explanation for the absence of a proximal external focus benefit may be derived from the nature of the task and the relationship between the performer and their equipment. In kayaking, the paddle is an implement to be actively manipulated to generate propulsion, whereas the boat is passive apparatus to be propelled. The performer is fitted to the kayak, which only moves whilst conjoined to the kayaker. A focus on the boat may therefore equate to a more proximal focus than one on the paddle. In effect, it may act as a quasi-internal focus due to the connected nature and relationship between kayak and kayaker.

It is also possible that a focus on the boat may have constrained the participants’ ability to sub-consciously balance and orientate the craft. Further, this boat focus may have diverted critical attentional resources from both effective propulsion and the goal of the activity. This then may have rendered the proximal focus more disruptive even compared to the participants’ self-selected foci in the control condition.

The apparent disparity between the results of the present study (proximal focus disrupted performance relative to a control condition) and previous work which has not found a difference between a proximal focus and a control condition (e.g., Banks et al., 2020) suggests that a proximal–distal dichotomy might not always be nuanced enough to explain differences in external focus effects. As well as its distality, it could be that the specific focus point used is also important. For example, in some instances a very proximal external focus could act similarly to an internal focus, especially when the proximal focus is on passive apparatus to which the performer is attached. In a case such as this, it seems possible that a proximal external focus may concentrate attention on movement and balance (e.g., of the boat) that would otherwise be under the control of automatic processes.

It is worth considering what the optimal focus might be for performers of varying levels of expertise in a continuous skill. In this study we chose the finish as the distal focal point, but this is only one of a number of potential points between the boat (very proximal) and the finish (very distal). It is an open question what the optimal focal point might be in any given activity and how to optimize it. This could be either more proximally to the performer, or more distally to the goal, or somewhere in between, particularly in disciplines where the finish or end point is much further away from the start. The optimal focal point may not even remain the same throughout the performance of a continuous skill, it might switch between points. It also seems likely it may differ depending on the activity, the complexity of the environment being moved through and at what point in the activity a participant is.

With regard to expertise, one potentially important difference between the two study groups in the present work is the amount and structure of their training, combined with its intended purpose. For the recreational kayakers, their motivation is to be better (and faster) paddlers so they can enjoy their activity more. For the K1 sprinters, it is to perform to the highest standard and achieve the best times and positions possible in races. As a result, the training the K1 sprinters undertake is structured with that goal in mind. The intention is to optimise their performance via a dedicated and defined training schedule, designed to help them go as fast as possible in races. Conversely, the recreational paddlers do not train to race and are therefore less likely to be paddling as close to their potential maximum speed. This means that a generally beneficial intervention, such as a distal focus, may have a more immediate and discernible effect on those performers with the most room to develop.

It is possible that the high and specific levels of training and reproducible skill of the K1 racers, meant that when tested in an applied skill in the environment in which they routinely perform it, improvements in performance might have been more difficult to elicit. This would be consistent with previous work, such as a study by Maloney and Gorman (2021), which found that external focus instructions did not result in differences in performance for skilled swimmers. However, their kinematic and kinetic measurements showed an external focus of attention did have discernible effects which resulted in superior self-organisation. In the present study, no such measurements were taken, though Maloney and Gorman’s work shows that it is possible for attentional focus instructions to have beneficial effects in skilled performers which are not reflected in immediately measurable performance outcomes. It could be the case that in experienced athletes, with high-level and tightly defined training, improvements in performance might be more difficult to detect. It is also possible that such advantages may take longer to become apparent, due to their already well-optimised technique. The present work shows that even with highly trained athletes, it is possible to disrupt performance within a single session via instructions to maintain a proximal external focus.

The results presented here are compatible with the idea that there may not necessarily be a difference between expert and recreational performers in terms of the effects of focus distance in continuous skills. Rather, it may be that the effects are more subtle in expert participants and not immediately discernible in terms of performance. It is also possible that the specific relationship between performer and equipment may exacerbate some attentional focus effects. The fact that an unfamiliar distal focus resulted in a sprint performance that matched the K1 racers’ well-practiced attentional focus in just one session is notable. If it was the case that participants already had the ideal/most appropriate/optimal focus, then any different, and therefore less ideal, focus would be expected to be disruptive to their performance. Future work could investigate whether a longer period of training using a distal focus might result in performance benefits. In activities where fractions of a second and other marginal gains make the difference between winning and losing, success and failure, such interventions may be critical.

## Footnotes

1 An open skill has variables in the performance environment which are partially or totally beyond the performer’s control e.g., weather, water conditions, terrain, other participants. The greater the speed and magnitude of variation the more open the skill.
